# The role of C-reactive protein as a prognostic marker in COVID-19

**DOI:** 10.1093/ije/dyab012

**Published:** 2021-03-03

**Authors:** Dominic Stringer, Philip Braude, Phyo K Myint, Louis Evans, Jemima T Collins, Alessia Verduri, Terry J Quinn, Arturo Vilches-Moraga, Michael J Stechman, Lyndsay Pearce, Susan Moug, Kathryn McCarthy, Jonathan Hewitt, Ben Carter, COPE Study Collaborators, Eilidh Bruce, Alice Einarsson, Aine McGovern, Carly Bisset, Ross Alexander, Giovanni Guaraldi, Caroline Murphy, Joanna Kelly, Tarik El Jichi Mutasem, Sandeep Singh, Dolcie Paxton, Will Harris, James Hesford, Mark Holloway, Emma Mitchel, Frances Rickard, Norman Galbraith, Emma Bhatti, Jenny Edwards, Siobhan Duffy, Fenella Barlow-Pay, Madeline Garcia, Shefali Sangani, Thomas Kneen, Thomas Lee, Angeline Price, Charlotte Davey, Sheila Jones, Kiah Lunstone, Alice Cavenagh, Charlotte Silver, Thomas Telford, Rebecca Simmons

**Affiliations:** 1 Department of Biostatistics and Health Informatics, Institute of Psychiatry, Psychology & Neuroscience, King's College London, London, UK; 2 North Bristol NHS Trust, UK; 3 Institute of Applied Health Sciences, University of Aberdeen; 4 Ysbyty Gwynedd, Bangor; 5 Ysbyty Ystrad Fawr, Aneurin Bevan University Health Board; 6 Hospital of Modena Policlinico, Italy; 7 Institute of Cardiovascular and Medical Sciences, University of Glasgow; 8 Department of Ageing and Complex Medicine, Salford Royal NHS Foundation Trust, Salford, University of Manchester, Manchester, UK; 9 Department of Surgery, University Hospital of Wales, Cardiff, UK; 10 Department of Colorectal Surgery, Salford Royal NHS Foundation Trust, Manchester, UK; 11 Department of Surgery, Royal Alexandra Hospital, Paisley, UK; 12 Department of Surgery, North Bristol NHS Trust, Bristol, UK; 13 Cardiff University and Aneurin Bevan University Health Board

**Keywords:** CRP, COVID-19, bimodal, trimodal, mortality, prognostic marker, mixture model

## Abstract

**Background:**

C-reactive protein (CRP) is a non-specific acute phase reactant elevated in infection or inflammation. Higher levels indicate more severe infection and have been used as an indicator of COVID-19 disease severity. However, the evidence for CRP as a prognostic marker is yet to be determined. The aim of this study is to examine the CRP response in patients hospitalized with COVID-19 and to determine the utility of CRP on admission for predicting inpatient mortality.

**Methods:**

Data were collected between 27 February and 10 June 2020, incorporating two cohorts: the COPE (COVID-19 in Older People) study of 1564 adult patients with a diagnosis of COVID-19 admitted to 11 hospital sites (test cohort) and a later validation cohort of 271 patients. Admission CRP was investigated, and finite mixture models were fit to assess the likely underlying distribution. Further, different prognostic thresholds of CRP were analysed in a time-to-mortality Cox regression to determine a cut-off. Bootstrapping was used to compare model performance [Harrell’s C statistic and Akaike information criterion (AIC)].

**Results:**

The test and validation cohort distribution of CRP was not affected by age, and mixture models indicated a bimodal distribution. A threshold cut-off of CRP ≥40 mg/L performed well to predict mortality (and performed similarly to treating CRP as a linear variable).

**Conclusions:**

The distributional characteristics of CRP indicated an optimal cut-off of ≥40 mg/L was associated with mortality. This threshold may assist clinicians in using CRP as an early trigger for enhanced observation, treatment decisions and advanced care planning.

**Key Messages**

- C-reactive protein (CRP) has been used inconsistently both in patient management and as a prognostic marker during COVID-19.
- Admission elevated CRP for patients with COVID-19 was associated with increased inpatient mortality and was indicative of disease severity at admission.
- The distribution of CRP at admission was found to be bimodally distributed, and a CRP ≥40 mg/L was the optimal threshold of increased risk of mortality.
- Admission CRP ≥40 mg/L may be used by treating clinicians as an early warning for enhanced care and patient-centred decision making.

## Introduction

Elevated levels of serum C-reactive protein (CRP) have been observed in patients with COVID-19 and used to assist with triage, diagnostics and prognostication.^1^^,^^2^ CRP is a non-specific acute phase protein that is produced by hepatocytes and elevated in acute infection or inflammation.^3^ Secretion begins 4–10 h after an inflammatory insult and peaks at 48 h, with a short half-life of 19 h. Crucially, it may be elevated before a patients’ vital signs are affected or leukocytes are raised.^3^ The profile of this biomarker has made CRP useful and routinely available in clinical medicine for diagnostics.

CRP can be used to assist with differentiation between viral and bacterial infections, for example, influenza produces a mean CRP level of 25.65 mg/L [95% confidence interval (CI) 18.88 to 32.41 mg/L] versus bacterial pneumonia which produces a mean CRP level of 135.96 mg/L (95% CI 99.38 to 172.54 mg/L).^4^ In COVID-19, a CRP level of ≥4 mg/L has been shown to be useful for triaging suspected cases when comparing polymerase chain reaction (PCR)-positive patients versus negative controls who have presented to a fever clinic with respiratory symptoms or a high temperature [odds ratio (OR) 4.75; 95% CI 3.28 to 6.88].^5^

However, debate remains over the utility of CRP as a prognostic marker for patients admitted to hospital with COVID-19. In a recent systematic review, 10 of the 22 included COVID-19 prognostic models treated CRP either as a factor or covariate.^6^ Most these studies used CRP with a binary threshold; proposed values to predict inpatient mortality varied from ≥10 mg/L to ≥76 mg/L. In addition to a binary threshold, CRP has been examined in a trichotomized model with the two thresholds at ≥40 mg/L and ≥100 mg/L.^7^ A lower cut-off of ≥20.44 mg/L was used as a threshold for related lung injury,^8^ and >32.5 mg/L was found to offer 80% predictive power for a person needing mechanical ventilation.^9^ The studies adjusted for admission CRP as a covariate to account for baseline disease severity have assumed a linear or natural logarithm transformation [Ln(_CRP_)] relationship with outcome.^10^^,^^11^ Although using CRP in a continuous manner may offer an improved understanding of the contribution of CRP within each analysis, it does not allow CRP to be used by clinical teams to guide management of patients with COVID-19.

Whilst CRP has been argued as an important marker of disease progression in COVID-19,^6^, its distribution has never been explored to understand whether distinct patterns exist in a heterogeneous population. The use of CRP as a biomarker in COVID-19 may present a quick and accessible tool in clinical management, trigger longer periods of enhanced observation, provide information around likely disease progression and assist with early therapeutic, ventilation and palliative care discussions.

The aim of this study is to examine the distribution of CRP at hospital admission, and objectives are to: (i) assess CRP as a prognostic bimodal or trimodal distribution; (ii) propose and compare the categorization of CRP as a prognostic marker to either a linear or a log-linear measure of CRP.

## Methods

Permission to conduct this study was granted in the UK by the Health Research Authority (20/HRA/1898) and in Italy by the ethics committee of University Hospital of Modena Policlinico (369/2020/OSS/AOUMO). Written consent was not required from participants as per ethical review.

### Study design

This observational study used two cohorts at different time points to examine the contribution of CRP to clinical outcomes. This study has been reported in accordance with the STROBE statement.^12^

### Settings

Thirteen hospital sites participated, 12 from the UK and one from Italy. All were acute hospitals directly admitting patients with suspected or confirmed COVID-19.

### Participants

#### Original cohort (cohort 1)

Participants in Cohort 1 were included as part of the COPE study (COVID in Older People study) as reported in the paper by Hewitt *et al.*^13^^,^^14^ Briefly, this was a European multicentre observational study recruiting 1564 hospitalized adults between 27 February and 28 April 2020 with either SARS-CoV-2 viral polymerase chain reaction (PCR) confirmed disease (95.9%) or clinically diagnosed (4.1%) COVID-19. Any patient aged 18 years or older admitted to the participating hospitals with a diagnosis of COVID-19 was included. The study found frailty was associated with longer hospital stay, and a better predictor of mortality as an inpatient, and at Day 7, than age or comorbidity alone.

#### Validation cohort (cohort 2)

Cohort 2 consisted of an additional 271 patients recruited between 29 April and 10 June 2020 from a combination of six of Cohort 1’s hospitals plus two additional recruiting hospitals. All patients were SARS-CoV-2 viral PCR-positive.

### Variables

A prognostic threshold for CRP was needed within the COPE protocol (March 2020). The limited literature available early in the pandemic included a case series of 73 patients with COVID-19 presenting with a mean CRP level of 51.4 mg/L [standard deviation (SD) 41.8].^1^ Based on this paper, and proposed by the clinical experience of the authors who delivered acute care, a dichotomous threshold was chosen with <40 mg/L (lower admission CRP), and ≥40 mg/L (CRP-elevated, indicating increased disease severity^14^).

### Data sources

CRP was measured at hospital admission and transcribed from patients’ medical records. There was no attempt to standardize the CRP assay between sites. A standardized case reporting form was used for all hospital sites. Data were transferred to King’s College London in anonymous format for statistical analysis.

### Graphical data analysis

Using the test cohort, the distribution of CRP was examined graphically and stratified by age. Finite bivariate and trivariate Gaussian mixture models were fit to CRP, representing two and three latent classes, respectively. The theoretical distribution from these models was compared with the empirical data and the threshold between the two and three classes was examined. The normality assumptions were assessed visually.

### Statistical analysis

#### Primary analysis: mixture modelling analysis

The empirical data from the test cohort were fit to a Gaussian mixture model with one, two or three components using an expectation-maximization algorithm (to refine the starting values) then maximum likelihood estimation (Stata routine ‘*fmm’*). The models were compared using the Akaike information criterion (AIC) and the thresholds were determined by the posterior probability of belonging to the two or three class models.

#### Secondary analysis: prognostic modelling analysis

To assess differing thresholds for CRP as a prognostic factor of outcome, a series of mixed-effects multivariable Cox proportional hazards models for time to mortality were fit, in a method consistent with the COPE study primary analysis.^13^ The model was adjusted for elevated CRP using a level of ≥40 mg/L, in addition to: patient age group (<65, 65–79, ≥80 years old), sex, diabetes (yes/no), hypertension (yes/no), coronary artery disease (yes/no) and kidney disease [estimated glomerular filtration rate (eGFR) <60 ml/min/1.73m^2^]. Dichotomized thresholds of CRP were compared within a range of 10 mg/L to 100 mg/L in 5-mg/L intervals (≥10 mg/L, ≥15 mg/L, etc). Model performance was evaluated and compared using Harrell’s C and the AIC.^15^ We compared the dichotomized thresholds against linear CRP and Ln(_CRP_) (as CRP is known to be skewed) as benchmarks of performance. This method was chosen as dichotomizing results can lead to a loss of information, resulting in a lower predictive power compared with using a continuous measure.^16^ Bootstrapping was used to construct 95% percent confidence intervals for differences in model performance between the best-fitting models. Bootstrapping was stratified by site with 1000 replications for each comparison. A complete case analysis was used in all cases due to negligible missing data (<4%).

### Validation cohort (cohort 2)

To provide an indication of whether the original results from Cohort 1 were likely to be replicable to a wider group of patients with COVID-19, the analysis was repeated on an independent validation sample (Cohort 2). Using the validation cohort, two-class and three-class mixture models were estimated using the empirical data without restriction. On evidence of overfitting, to assess the additional benefit of a very elevated category for CRP, the validation cohort was fitted using a three-class mixture model, with the class-two mean fixed using the validation cohort two-class mixture model mean.

### Comparison of the prognostic effect of CRP

Using a mixed-effect multivariable Cox regression, the effect of elevated CRP will be reported using a adjusted hazards ratio (aHR), alongside the respective 95% confidence interval (95% CI), for a linear CRP, Ln(_CRP_).

## Results

The study included 1835 patients across Cohorts 1 and 2, who were drawn from 12 hospitals in the UK and one from Italy. Of the total study participants, 26.4% (*n* = 484) died in-hospital, varying between sites from 13.3% to 42.9%. A comparison for those who died in hospital was carried out in Table 1, split into Cohort 1 (*n* = 1564) and Cohort 2 (*n* = 271). In Cohort 1, 27.2% died and the median CRP level for those who died was 115 mg/L (interquartile range: 63 mg/L-191 mg/L) compared with 69 mg/L (29 mg/L–140 mg/L) among those who survived. For patients with CRP ≥40 mg/L, mortality was 31.9% compared with 15.0% for patients with CRP <40 mg/L. Median follow-up time (time to mortality or discharge) was 13 days (6–22 days).

**Table 1:** Descriptive characteristics for Cohort 1 and 2 samples with comparison by in-hospital mortality

	Cohort 1 (Original)			Cohort 2 (Validation)		
	All patients (*n* = 1564)	Dead (*n* = 425)	Alive (*n* = 1139)	All patients (*n* = 271)	Dead (*n* = 59)	Alive (*n* = 212)
**Sites**						
Hospital A	115 (7·4%)	15 (13·0%)	100 (87·0%)	25 (9·2%)	4 (16·0%)	21 (84·0%)
Hospital B	50 (3·2%)	14 (28·0%)	36 (72·0%)	0 (0%)	0 (0%)	0 (0%)
Hospital C	153 (9·8%)	34 (22·2%)	119 (77·8%)	0 (0%)	0 (0%)	0 (0%)
Hospital D	43 (2·7%)	10 (23·3%)	33 (76·7%)	9 (3·3%)	0 (0·0%)	9 (100·0%)
Hospital E	123 (7·9%)	15 (12·2%)	108 (87·8%)	58 (21·4%)	9 (15·5%)	49 (84·5%)
Hospital F	154 (9·8%)	23 (14·9%)	131 (85·1%)	15 (5·5%)	4 (26·7%)	11 (73·3%)
Hospital G	112 (7·2%)	36 (32·1%)	76 (67·9%)	0 (0%)	0 (0%)	0 (0%)
Hospital H	246 (15·7%)	108 (43·9%)	138 (56·1%)	0 (0%)	0 (0%)	0 (0%)
Hospital I	380 (24·3%)	126 (33·2%)	254 (66·8%)	62 (22·9%)	12 (19·4%)	50 (80·7%)
Hospital J	179 (11·5%)	43 (24·0%)	136 (76·0%)	13 (4·8%)	3 (23·1%)	10 (76·9%)
Hospital K	9 (0·6%)	1 (11·1%)	8 (88·9%)	4 (1·5%)	1 (25·0%)	3 (75·0%)
Hospital L	0 (0%)	0 (0%)	0 (0%)	57 (21·0%)	14 (24·6%)	43 (75·4%)
Hospital M	0 (0%)	0 (0%)	0 (0%)	28 (10·3%)	12 (42·9%)	16 (57·1%)
**Age, years**						
<65	488 (31·2%)	55 (11·3%)	433 (88·7%)	61 (22·5%)	6 (9·8%)	55 (90·2%)
65–79	535 (34·2%)	168 (31·4%)	367 (68·6%)	85 (31·4%)	14 (16·5%)	71 (83·5%)
≥80	541 (34·6%)	202 (37·3%)	339 (62·7%)	124 (45·8%)	38 (30·7%)	86 (69·4%)
Missing	0 (0·0%)	0 (0·0%)	0 (0·0%)	1 (0·4%)	1 (100·0%)	0 (0·0%)
**Sex**						
Female	661 (42·3%)	170 (25·7%)	491 (74·3%)	134 (49·5%)	29 (21·6%)	105 (78·4%)
Male	903 (57·7%)	255 (28·2%)	648 (71·8%)	136 (50·2%)	30 (22·1%)	106 (77·9%)
Missing	0 (0%)	0 (0%)	0 (0%)	0 (0%)	0 (0%)	1 (100.0%)
**Smoking status**						
Never smokers	814 (52·0%)	205 (25·2%)	609 (74·8%)	132 (48·7%)	24 (18·2%)	108 (81·8%)
Ex-smokers	603 (38·6%)	185 (30·7%)	418 (69·3%)	91 (33·6%)	25 (27·5%)	66 (72·5%)
Current smokers	121 (7·7%)	26 (21·5%)	95 (78·5%)	18 (6·6%)	2 (11·1%)	16 (88·9%)
Missing	26 (1·7%)	9 (34·6%)	17 (65·4%)	30 (11·1%)	8 (26·7%)	22 (73·3%)
**Diabetes**						
No	1144 (73·1%)	295 (25·8%)	849 (74·2%)	204 (75·3%)	41 (20·1%)	163 (79·9%)
Yes	415 (26·5%)	128 (30·8%)	287 (69·2%)	66 (24·4%)	18 (27·3%)	48 (72·7%)
Missing	5 (0·3%)	2 (40·0%)	3 (60·0%)	1 (0·4%)	0 (0·0%)	1 (100·0%)
**Hypertension**						
No	755 (48·3%)	184 (24·4%)	571 (75·6%)	126 (46·5%)	22 (17·5%)	104 (82·5%)
Yes	804 (51·4%)	238 (29·6%)	566 (70·4%)	145 (53·5%)	37 (25·5%)	108 (74·5%)
Missing	5 (0·3%)	3 (60·0%)	2 (40·0%)	0 (0%)	0 (0%)	0 (0%)
**Coronary artery disease**						
No	1214 (77·6%)	290 (23·9%)	924 (76·1%)	220 (81·8%)	43 (19·6%)	177 (80·5%)
Yes	345 (22·1%)	132 (38·3%)	213 (61·7%)	51 (18·8%)	16 (31·4%)	35 (68·6%)
Missing	5 (0·3%)	3 (60·0%)	2 (40·0%)	0 (0%)	0 (0%)	0 (0%)
**Increased C-reactive protein (≥40 mg/L)**						
No	439 (28·1%)	66 (15·0%)	373 (85·0%)	96 (35·4%)	10 (10·4%)	86 (89·6%)
Yes	1125 (71·9%)	359 (31·9%)	766 (68·1%)	161 (59·4%)	46 (28·6%)	115 (71·4%)
Missing	32 (2·0%)	12 (37·5%)	20 (62·5%)	14 (5·2%)	3 (21·4%)	11 (78·6%)
**Impaired renal function (eGFR^a^ <60 mL/min per 1·73 m^2^)**						
No	980 (63·7%)	202 (20·6%)	778 (79·4%)	132 (48·7%)	20 (15·2%)	112 (84·9%)
Yes	570 (36·4%)	217 (38·1%)	353 (61·9%)	81 (29·9%)	25 (30·9%)	56 (69·1%)
Missing	14 (0·9%)	6 (42·9%)	8 (57·1%)	58 (21·4%)	14 (24·1%)	44 (75·9%)
**Clinical Frailty Scale (1-9)**						
1: Very fit	91 (5·8%)	7 (7·7%)	84 (92·3%)	11 (4·1%)	1 (9·1%)	10 (90·9%)
2: Fit	197 (12·6%)	22 (11·2%)	175 (88·8%)	22 (8·1%)	1 (4·6%)	21 (95·5%)
3: Managing well	287 (18·4%)	55 (19·2%)	232 (80·8%)	37 (13·7%)	7 (18·9%)	30 (81·1%)
4: Vulnerable	185 (11·8%)	52 (28·1%)	133 (71·9%)	25 (9·2%)	5 (20·0%)	20 (80·0%)
5: Mildly frail	182 (11·6%)	50 (27·5%)	132 (72·5%)	38 (14·0%)	6 (15·8%)	32 (84·2%)
6: Moderately frail	251 (16·0%)	84 (33·5%)	167 (66·5%)	51 (18·8%)	11 (21·6%)	40 (78·4%)
7: Severely frail	260 (16·6%)	96 (36·9%)	164 (63·1%)	61 (22·5%)	20 (32·8%)	41 (67·2%)
8: Very severely frail	79 (5·1%)	44 (55·7%)	35 (44·3%)	5 (1·9%)	3 (60·0%)	2 (40·0%)
9: Terminally ill	27 (1·7%)	12 (44·4%)	15 (55·6%)	4 (1·5%)	1 (25·0%)	3 (75·0%)
Missing	5 (0·3%)	3 (60·0%)	2 (40·0%)	17 (6·3%)	4 (23·5%)	13 (76·5%)
**Median CRP^b^ (mg/L) (lower and upper quartile)**						
	80·5 (36–154)	115 (63-191)	69 (29–140)	65 (20-117)	86 (48–173·5)	53 (16–109)

a Estimated glomerular filtration rate.

b C-reactive protein.

Cohort 2 experienced 21.8% mortality. Among those who died, median CRP level was 86 mg/L (48 mg/L-173.5 mg/L) compared with 53 mg/L (16 mg/L-109 mg/L) among those who survived. For patients with CRP ≥40 mg/L, mortality was 28.6% compared with 10.4% for patients with CRP <40 mg/L. The median follow-up time (time to death or discharge) was 10 days (5–18 days).

### Results of cohort 1 (n = 1564)

#### Distribution of CRP

**Figure 1:**
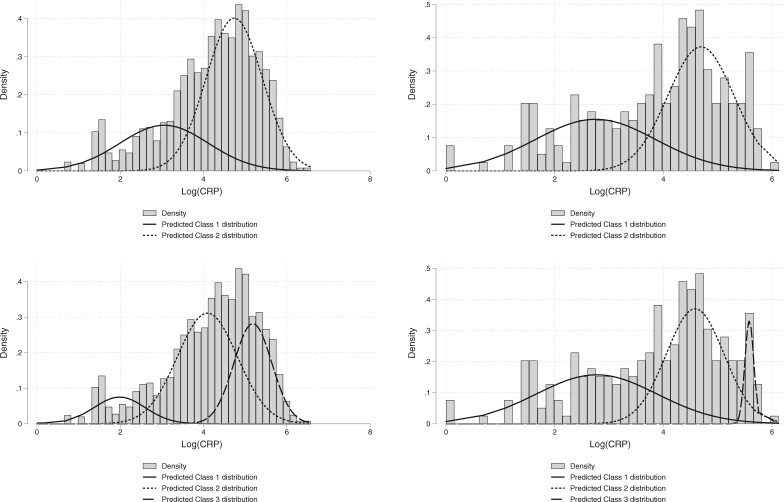
Distribution of C-reactive protein (CRP) in the original cohort (left panel) and the validation cohort (right panel), overlaid predicted distributions from a two-class (upper row), compared with three-class (lower row) finite mixture models

On graphical examination of the distribution of Ln(_CRP_), it exhibited negative skew, with two ‘peaks’ suggestive of a bimodal distribution, see Figure 1, Plot (i), and Figure S1, available as Supplementary data at *IJE* online, Plots (i, ii). The distribution of Ln(_CRP_) was observed in age-stratified groups of <65, 65–79, and ≥80 years. On inspection, there was no difference between the distribution age-stratified or the complete dataset.

### Primary analysis: mixture modelling analysis

Following the two suggested peaks in the examination of the Ln(_CRP_) distribution, a two-latent class finite mixture model was fitted. It appeared to graphically fit the data when examined against the empirical distribution in Figure 1, Plot (i). This was supported by a comparison with the one-class (or null) model, which displayed a higher AIC (4739 compared with 4524). The simple threshold at which the predicted probability of belonging to a two-class model being greater than one-class was 38 mg/L. This will be implemented as ≥ 40 mg/L herein, to account for the imprecision of the measurement of CRP and also for ease of recall in a busy clinical setting.

The three-class finite mixture model fit slightly better than the two-class finite mixture model (AIC of 4484), with probability of class-one membership highest between range 0–14 mg/L, class-two between 15–120 mg/L and class-three for values of CRP ≥120 mg/L, see Figure 1, Plot (iii).

The primary analysis proposed a single optimal threshold of CRP ≥40 mg/L to indicate elevated CRP.

### Secondary analysis: prognostic modelling

The time-to-mortality analysis included 1502 participants (96%) in the complete case population. A cut-off of ≥65 mg/L appeared to fit best in the sample on all measures (Harrell’s C statistic of 0.7068, AIC of 5124) (Table 2) after fitting different binary categorizations of CRP in a Cox model for time to mortality. Differences in measures of goodness of fit were small, especially between cut-offs in the range of ≥40 mg/L to ≥90 mg/L. CRP as a continuous Ln(_CRP_) measure performed considerably better (Harrell’s C statistic of 0.7157, AIC of 5001) and with little improvement on this using a linear scale (Harrell’s C statistic of 0.7040, AIC of 5024). Regarding bootstrapped differences in the measures of goodness of fit between a cut-off of ≥40 mg/L and the marginally better performing cut-off of ≥65 mg/L, no difference in performance was seen with 95% CI for all measures (Table 3). There was evidence that cut-offs of both ≥40 mg/L and ≥65 mg/L outperformed a cut-off of ≥10 mg/L, the upper limit of the normal range for CRP.^17^ It should be noted that Ln(_CRP_) was the optimal parameterization compared with either ≥40 mg/L (−135.1 AIC, bootstrapped 95% CI -210.4 to −65.1) or ≥65 mg/L (−123.5 AIC, bootstrapped 95% CI −197.6 to −55.8).

**Table 2:** Performance of different cut-offs/parametrizations of C-reactive protein (CRP) in a Cox model for time to mortality

CRP (mg/L) parametrization	Cohort 1 (Original)			Cohort 2 (Validation)		
	*N* below cut-off (%)	Harrell’s C statistic	AIC	*N* below cut off (%)	Harrell’s C statistic	AIC
Null (CRP not included)	NA	0.6592	5224.41	NA	0.6816	428.58
CRP ≥ 10	132 (8.6%)	0.6697	5174.10	39 (15.2%)	0.6811	430.58
CRP ≥ 15	190 (12.4%)	0.6797	5159.14	56 (21.8%)	0.6995	429.22
CRP ≥ 20	230 (15.0%)	0.6858	5148.08	66 (25.7%)	0.7024	426.59
CRP ≥ 25	279 (18.2%)	0.6930	5144.01	75 (29.2%)	0.7055	427.83
CRP ≥ 30	326 (21.3%)	0.6953	5143.46	83 (32.3%)	0.7044	427.44
CRP ≥ 35	381 (24.9%)	0.6963	5141.06	91 (35.4%)	0.7145	425.61
CRP ≥ 40	439 (28.7%)	0.7024	5136.10	96 (37.4%)	0.7187	424.35
CRP ≥ 45	486 (31.7%)	0.7055	5132.53	103 (40.1%)	0.7015	427.18
CRP ≥ 50	530 (34.6%)	0.7059	5126.61	111 (43.2%)	0.6974	428.68
CRP ≥ 55	569 (37.1%)	0.7025	5130.50	120 (46.7%)	0.6900	428.85
CRP ≥ 60	605 (39.5%)	0.7064	5127.69	125 (48.6%)	0.6926	428.16
CRP ≥ 65	648 (42.3%)	0.7068	5124.45	129 (50.2%)	0.6867	428.47
CRP ≥ 70	687 (44.8%)	0.7033	5131.22	135 (52.5%)	0.6895	428.32
CRP ≥ 75	727 (47.5%)	0.7006	5131.79	139 (54.1%)	0.6879	428.87
CRP ≥ 80	766 (50.0%)	0.7005	5133.82	145 (56.4%)	0.6853	429.49
CRP ≥ 85	804 (52.5%)	0.7021	5135.11	155 (60.3%)	0.6887	429.16
CRP ≥ 90	834 (54.4%)	0.7001	5138.43	161 (62.6%)	0.6816	430.34
CRP ≥ 95	863 (56.3%)	0.6975	5142.08	169 (65.8%)	0.6828	429.98
CRP ≥ 100	887 (57.9%)	0.7010	5137.48	173 (67.3%)	0.6890	429.69
CRP (linear)	NA	0.7040	5024.81	NA	0.6992	426.42
log(CRP)	NA	0.7157	5001.00	NA	0.7014	426.51

Number of cases defined as not elevated CRP.

NA, not available.

**Table 3:** Bootstrapped differences in model performance of Cox model for time to mortality for different C-reactive protein (CRP) parameterizations using Cohort 1 (Original Cohort)

Model comparison	Difference in	Coefficient	Bias	Standard error	95% CI	
≥10 compared with ≥40	Harrell's C statistic	−0.033	0.003	0.007	−0.044	−0.016
	AIC^+^	38.002	−0.326	12.591	14.250	64.258
≥65^a^ compared with ≥40	Harrell's C statistic	0.004	0.002	0.007	−0.006	0.020
	AIC	−11.655	0.260	11.400	−32.978	11.775
Linear CRP compared with ≥40	Harrell's C statistic	0.002	0.003	0.008	−0.010	0.020
	AIC	−111.289	102.784	15.212	−41.031	19.531
Ln_(CRP)_ compared with ≥40	Harrell's C statistic	0.013	0.002	0.006	0.004	0.028
	AIC	−135.105	−0.857	37.105	−210.386	−65.123
≥10 compared with ≥65^a^	Harrell's C statistic	−0.037	0.001	0.010	−0.058	−0.018
	AIC	49.657	−0.585	15.262	21.293	79.554
Linear CRP compared with ≥65^a^	Harrell's C statistic	−0.003	0.001	0.007	−0.016	0.012
	AIC	−99.633	102.470	13.781	−25.825	29.435
Log_(CRP)_ compared with ≥65^a^	Harrell's C statistic	0.009	0.000	0.006	−0.002	0.021
	AIC	−123.450	−1.117	36.286	−197.611	−55.831
Linear CRP compared with ≥10	Harrell's C statistic	0.034	0.000	0.010	0.018	0.056
	AIC	−149.291	103.429	15.581	−77.820	−18.453
Log_(CRP)_ compared with ≥10	Harrell's C statistic	0.046	−0.001	0.009	0.028	0.063
	AIC	−173.107	−0.531	38.325	−253.435	−99.153
Log _(CRP)_ compared with Linear CRP	Harrell's C statistic	0.012	−0.001	0.004	0.003	0.019
	AIC	−23.816	0.618	9.527	−41.730	−4.322

a A threshold of ≥65 has been included as a comparison with ≥40.

### Results of cohort 2 (n = 271)

#### Distribution of CRP

Cohort 2 included 271 new patients from eight hospital sites: 85 (31.4%) were fully independent, recruited from two new hospital sites; 186 were pseudo-independent, being newly recruited patients from original hospital sites in Cohort 1. There was no difference in the demographics, comorbidities and distribution of CRP seen in Cohort 2 and Cohort 1 (Table 1).

#### Fitting finite mixture models

The empirical distribution of the Cohort 2 Ln(_CRP_) appeared, graphically, to have a reasonably similar pattern to Cohort 1, see Figure 1, Plot (ii). The two-class finite mixture model gave a consistent threshold (CRP ≥41 mg/L). The unrestricted three-class finite mixture model exhibited likely overfitting to the data on examination of the distributions. Inconclusive evidence for the additional second cut-off was found with the class three distribution entirely contained within class two, with a large variance. There was no additional benefit for fixing the central distribution mean and allowing the mixture proportion to vary, but this can be seen graphically in Figure 1, Plot (iv). The simple threshold between class one and class two was ≥41 mg/L.

The time-to-mortality analysis included 208 of the participants (77%) with complete data. Fitting different binary categorizations of CRP in a Cox model for time to mortality gave a CRP cut-off of ≥40 mg/L as the best fitting model (Harrell’s C statistic of 0.7187, AIC of 424), outperforming the Ln(_CRP_) model (Harrell’s C statistic of 0.7014, AIC of 427), see Table 2. There was no evidence of difference in performance between cut-offs of ≥65 mg/L and ≥40 mg/L, nor between ≥40 mg/L and Ln(_CRP_) on examination of bootstrapped 95% CI in Supplementary Table S1, available as Supplementary data at *IJE* online.

#### The prognostic effect of elevated CRP with prognostic properties

The aHRs for CRP ≥40 mg/L were 2.58 (95% CI 1.95 to 3.41) and 2.61 (95% CI 0.54 to 4.63) for Cohorts 1 and 2 and the estimate of CRP appeared stable (Supplementary Table S2, available as Supplementary data at *IJE* online). For comparison CRP ≥65 mg/L, the aHR was consistent in Cohort 1 (aHR = 2.48; 95% CI 1.96 to 3.14) but appeared unstable in Cohort 2 (aHR = 1.61; 95% CI 0.84 to 3.09). Using a cut-off of ≥40, the sensitivity, specificity, positive predictive value and negative predictive value were 0.84; 0.33; 0.32; and 0.85 for Cohort 1 and 0.82, 0.43, 0.29 and 0.90 for Cohort 2, respectively.

## Discussion

### Key results

CRP reasonably followed a bimodal distribution using data from two independent cohorts. There was inconclusive evidence of a trimodal distribution; although the AIC metric suggested it fit better, on graphical examination there appeared to be overfitting.

In an analysis of 1835 patients across 13 hospital sites using a binary cut-off for CRP as a prognostic factor of COVID-19, inpatient death appeared to have similar predictive power compared with treating it as a linear or Ln(_CRP_). In addition, a cut-off value to indicate disease severity is simpler to use in a clinical setting than a linear predictor. These findings support the use of a simple binary threshold for CRP in daily clinical medicine. These results are well aligned with many published analyses in COVID-19 which have already employed a binary cut-off.^4^^,^^18–20^

The bimodal distribution of CRP may reflect the presence of a latent class influence. Candidate variables for this latent class may include confounders that were not fully controlled for: chronic inflammatory conditions, genomic variation of the virus, genetic susceptibility of populations or other binary exposures such as Bacillus Calmette-Guérin (BCG) vaccination status.^21–23^

The association of higher CRP with worse outcomes may be due to the severity of the disease consistent with the ‘cytokine storm’ theory of COVID-19, where the innate immune system is activated releasing TNF-alpha, IL-6 and IL-1. Elshazli *et al*. found CRP to be a valid biomarker of death from COVID-19 when examining a range of haematological and immunological markers. IL-6 was found to be most predictive (OR = 13.87) of death, and CRP the next best marker (OR = 7.09).^24^ However, IL-6 is not routinely available to clinicians, but being linked to CRP as a trigger for its transcription makes CRP a better candidate tool for front-line hospital usage.^25^ In the same Elshazli paper, a threshold level of 38.2 mg/L was demonstrated to have the best sensitivity and specificity, which fits well with our findings; this was also found within a recent Cochrane Diagnostic Test Accuracy review.^26^ In addition, an elevated CRP may not be attributable to COVID-19 alone and may represent concomitant pathology such as secondary bacterial pneumonia. Although co-infection is well known in other viral respiratory illnesses, the rate in COVID-19 has been found to be far less, being present in around 5.9% of the general COVID-19 hospital population and 8.1% of those with critical illness.^18^

The data presented here support a single threshold, and whilst there was argument for competing cut-offs of ≥40, ≥65 or greater, the single cut-off is consistent with other studies.^8^^,^^24^ In addition, it would be clearer and safer to offer a conservative approach using the lower value of CRP, as a higher threshold may falsely reassure clinicians.

There is a need for simple tests to aid clinical management, as the behaviour of CRP in COVID-19 may provide useful immediate risk stratification as to who may have a poor outcome. The threshold of CRP ≥40 offered a high negative predictive value, so patients presenting with a low CRP are unlikely to exhibit disease progression, and high sensitivity analysis which might lead to opening discussions with patients and their carers about the possible course of the disease. This may assist with early resource planning around the potential for critical care support, and may help guide rapid safe discharge from acute hospitals.^5^ Although the results within this paper give a population-based cut-off, any interpretation and management plan must be made on an individual patient basis, with clinicians using CRP in context of clinical history, examination and investigation and noting that the threshold offered a low positive predictive value. Beyond clinical predictive value, this model may be useful for monitoring the outcomes of treatments, for example in a trial of tocilizumab, CRP monitoring was used as a marker of efficacy.^26^

### Strengths and limitations

This was a large study that included participants admitted to 13 hospital sites. The demographics, case mix and mortality are similar to other larger studies reported within the UK, increasing the findings’ generalizability.^20^ We have also shown good replication between the two UK-wide cohorts. However, caution should be given to the threshold reported for CRP, as studies identifying optimal cut-offs may be subject to selection bias and may not be replicable.^27^ Using a threshold of ≥40 offered a high sensitivity and negative predictive value but low positive predictive value.

A limitation of this study is that due to the urgent nature of research data collection in a pandemic, disease severity on admission was only assessed using CRP without collection of circulating lymphocytes, interleukin-6, procalcitonin, serum lactate and viral load, all of which may also contribute to disease severity.^28^

### Interpretation

A simple threshold ≥40 mg/L should be used within clinical practice to guide disease severity and likely disease progression. Future studies should analyse using this simple threshold.

### Generalizability

The impact of these findings support the routine assessment of serum CRP as an adjunct in the early diagnosis and assessment of illness severity of hospitalized patients with COVID-19. We recommend that CRP ≥40 mg/L on admission may indicate an increased risk of disease progression and death, and warrants an enhanced level of discussion and clinical support.

## Conclusions

We have demonstrated that CRP follows a bimodal distribution in hospitalized patients with COVID-19. This requires further exploration to discover the latent class effect of unobserved factors influencing the distribution of CRP. A CRP of ≥40 mg/L on admission to hospital should be seen as a reliable indicator of disease severity and increased risk of death. We recommend clinicians use this cut-off as a prognostic indicator only, in conjunction with an individualized clinical assessment, frailty assessment and incorporating a person’s wishes and values, to make early decisions about enhanced observation, critical care support and advanced care planning.

## Supplementary data

Supplementary data are available at *IJE* online.

## Data availability

Data are available on request from the corresponding author after submission of a statistical analysis plan, after approval from the COPE Study Investigators.

## Funding

This study received no specific funding. The study was partially supported through the NIHR Maudsley Biomedical Research Centre at the South London and Maudsley NHS Foundation Trust in partnership with King's College London (B.C.).

## Cope Study Collaborators

Aberdeen University: Dr Eilidh Bruce, Dr Alice Einarsson; Glasgow Royal Infirmary: Dr Aine McGovern; Inverclyde Royal Infirmary: Carly Bisset, Ross Alexander; Italy (University Hospital of Modena Policlinico): Professor Giovanni Guaraldi; King’s College London: Caroline Murphy, Joanna Kelly, Dr Roxanna Short;North Bristol Trust: Tarik El Jichi Mutasem, Sandeep Singh, Dolcie Paxton, Will Harris, Dr James Hesford, Dr Mark Holloway, Dr Emma Mitchel, Dr Frances Rickard; Royal Alexandra Hospital, Paisley: Norman Galbraith, Emma Bhatti, Jenny Edwards, Siobhan Duffy, Dr Fenella Barlow-Pay; Salford Royal Infirmary: Madeline Garcia, Shefali Sangani, Thomas Kneen, Thomas Lee, Angeline Price; Ysbyty Yystad Fawr: Dr Charlotte Davey, Ms Sheila Jones, Kiah Lunstone, Alice Cavenagh, Charlotte Silver, Thomas Telford, Rebecca Simmons.

## Author contributions

Concept of the study (B.C., P.B.); developed the protocol (B.C., D.S., P.B.); collected the data (P.B., P.M., L.E., J.C., V.A., T.Q., A.V., M.S., L.P., J.H., S.M., K.Mc.); analysed the data (D.S., B.C.); interpreted the findings (D.S., B.C., P.B.); drafted the initial manuscript (D.S., P.B., B.C.); all authors approved the final manuscript. B.C. is the guarantor of the study findings.

## Conflict of interest

None declared.

## Supplementary Material

dyab012_Supplementary_Data
