# Supplementary material for: The role of C-reactive protein as a prognostic marker in COVID-19
Source: Int J Epidemiol. 2021 Mar 3;50(2):420–9. doi: 10.1093/ije/dyab012 (PMC7989395; doi:10.1093/ije/dyab012)
Supplement: dyab012_Supplementary_Data [file dyab012_supplementary_data.docx]

**Supplementary Data**

Figure S1 - Distribution of CRP in the original Cohort (left panel) and the validation cohort (right panel); overlaid predicted distributions from a two-class (upper row), compared to three-class (lower row) finite mixture models.

| i) | ii) |
| --- | --- |
| 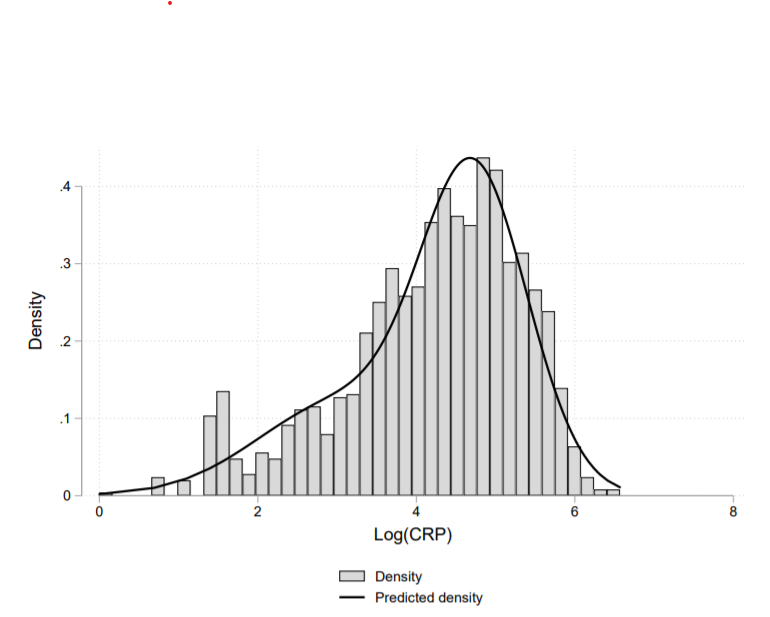 | 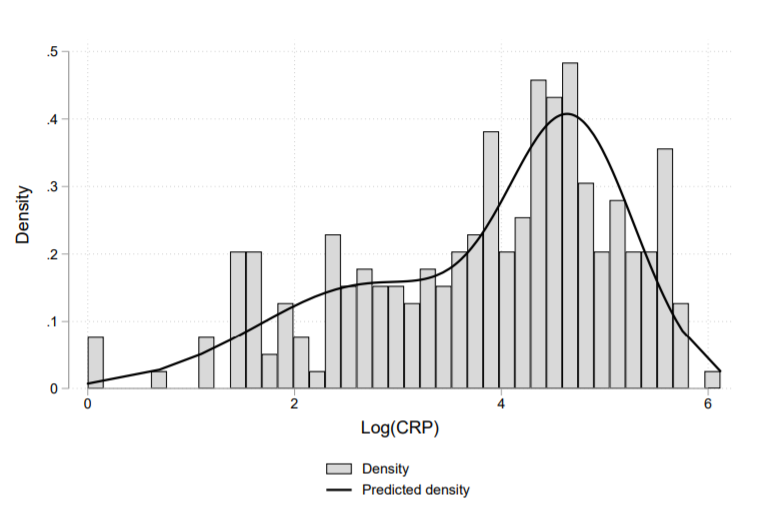 |
|  |  |
| iii) | iv) |
| 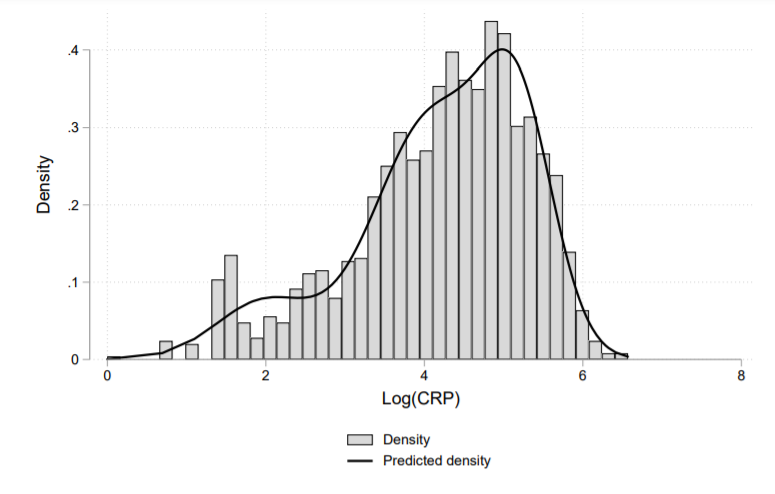 | 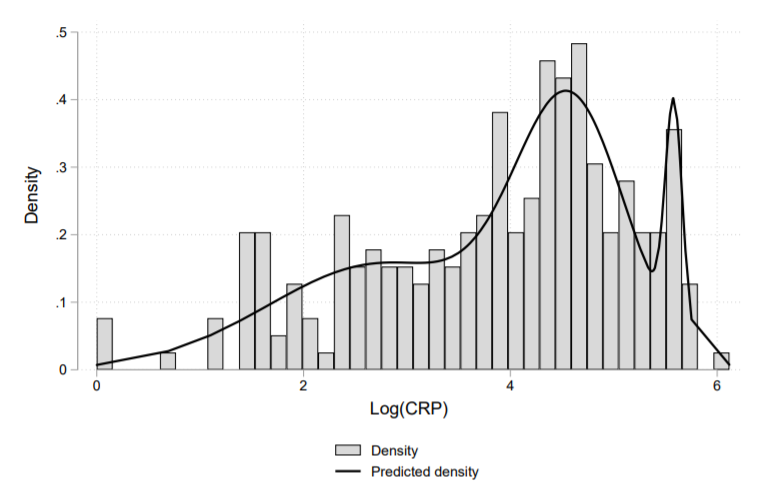 |

Note: Plot (i) show the empirical Original Data with an overlaid two-class mixture model;

Plot (ii) show the empirical Validation Data with an overlaid two-class mixture model;

Plot (iii) show the empirical Original Data with an overlaid three-class mixture model;

Plot (iv) show the empirical Validation Data with an overlaid three-class mixture model. The mean of the central distributed was fitted by the mean of the Original Cohort two-class mixture model.

Table S1 Bootstrapped differences in model performance of Cox model for time to mortality for different CRP parameterisations in Cohort 2 (Validation Cohort)

| Model comparison | Difference in: | Coef. | Bias | Standard error | 95% Percentile CI | |
| --- | --- | --- | --- | --- | --- | --- |
| ≥10 compared to ≥40 | Harrell's C | -0.038 | 0.008 | 0.019 | -0.069 | 0.005 |
|  | AIC | 6.230 | -0.432 | 5.272 | -3.044 | 17.376 |
| ≥65 compared to ≥40 | Harrell's C | -0.032 | 0.009 | 0.020 | -0.068 | 0.009 |
|  | AIC | 4.122 | -0.033 | 4.239 | -2.780 | 13.396 |
| Linear CRP compared to ≥40 | Harrell's C | 0.018 | -0.001 | 0.019 | -0.012 | 0.060 |
|  | AIC | -4.162 | 0.697 | 4.582 | -13.801 | 4.201 |
| Ln_(CRP)_ compared to ≥40 | Harrell's C | -0.017 | 0.006 | 0.013 | -0.040 | 0.011 |
|  | AIC | 2.166 | 0.381 | 2.884 | -2.225 | 9.540 |
|  |  |  |  |  |  |  |
| ≥10 compared to ≥65 | Harrell's C | -0.006 | -0.002 | 0.016 | -0.042 | 0.021 |
|  | AIC | 2.108 | -0.399 | 3.670 | -4.564 | 10.708 |
| Linear CRP compared to ≥65 | Harrell's C | 0.013 | -0.003 | 0.014 | -0.014 | 0.043 |
|  | AIC | -2.054 | 0.298 | 3.096 | -8.921 | 3.544 |
| Log_(CRP)_ compared to ≥65 | Harrell's C | 0.015 | -0.003 | 0.014 | -0.014 | 0.043 |
|  | AIC | -1.956 | 0.415 | 2.869 | -8.427 | 3.203 |
|  |  |  |  |  |  |  |
| Linear CRP compared to ≥10 | Harrell's C | 0.018 | -0.001 | 0.019 | -0.012 | 0.060 |
|  | AIC | -4.162 | 0.697 | 4.582 | -13.801 | 4.201 |
| Log_(CRP)_ compared to ≥10 | Harrell's C | 0.020 | -0.002 | 0.016 | -0.010 | 0.053 |
|  | AIC | -4.064 | 0.813 | 4.357 | -12.536 | 4.776 |
| Log _(CRP)_ compared to Linear CRP | Harrell's C | 0.002 | -0.001 | 0.011 | -0.020 | 0.023 |
|  | AIC | 0.099 | 0.116 | 2.339 | -4.333 | 5.272 |

Table S2 - Estimates for the competing models, presenting the Hazard Ratio (HR) and adjusted HR (aHR)

|  | Cohort 1 (Original) | | Cohort 2 (Validation) | | Total | |
| --- | --- | --- | --- | --- | --- | --- |
|  | Crude  HR (95% CI) | Adjusted  aHR^&^ (95% CI) | Crude  HR (95% CI) | Adjusted  aHR^&^ (95% CI) | Crude  HR (95% CI) | Adjusted  aHR^&^ (95% CI) |
| CRP^1^ | 1.32 (1.22,1.43) | 1.42 (1.31, 1.54) | 1.46 (1.15, 1.86) | 1.36 (1.01, 1.85) | 1.34 (1.24, 1.44) | 1.41 (1.31, 1.53) |
| Log(CRP) | 1.47 (1.32,1.64) | 1.65 (1.47, 1.85) | 1.42 (1.10,1.83) | 1.32 (0.98,1.78) | 1.46 (1.33,1.62) | 1.61 (1.45,1.79) |
| ≥40 | 2.20 (1.67,2.90) | 2.58 (1.95, 3.41) | 2.62 (1.31,5.21) | 2.61 (0.54,4.63) | 2.28 (1.77,2.94) | 2.56 (1.97,3.33) |
| ≥65^&&^ | 2.09 (1.66,2.63) | 2.48 (1.96, 3.14) | 1.81 (1.04,3.17) | 1.61 (0.84,3.09) | 2.05 (1.66,2.53) | 2.35 (1.89,2.92) |

^&^Notes: Adjusted Hazard ratio included covariates for: age; sex, diabetes (yes/no), hypertension (yes/no), coronary artery disease (yes/no), and kidney disease (eGFR <60 ml/min/1.73m2). All models included a random effect for site.

^&&^  CRP≥65 mg/L is presented as a comparison to ≥40 mg/L.

^1^CRP was first standardized.
